# Understanding the improvement mechanism of plasma etching treatment on oxygen reduction reaction catalysts

**DOI:** 10.1002/EXP.20230034

**Published:** 2023-11-14

**Authors:** Peng Rao, Yanhui Yu, Shaolei Wang, Yu Zhou, Xiao Wu, Ke Li, Anyuan Qi, Peilin Deng, Yonggang Cheng, Jing Li, Zhengpei Miao, Xinlong Tian

**Affiliations:** ^1^ School of Marine Science and Engineering Hainan Provincial Key Lab of Fine Chemistry School of Chemistry and Chemical Engineering Hainan University Haikou China; ^2^ Key Laboratory of Polyoxometalate and Reticular Material Chemistry of Ministry of Education School of Chemistry Northeast Normal University Changchun China; ^3^ National Energy Group Ledong Power Generation Co., Ltd Ledong China; ^4^ Laboratory for chemical technology Ghent University Gent Belgium

**Keywords:** activity improvement mechanism, electrocatalysts, oxygen reduction reaction, plasma etching treatment

## Abstract

Plasma etching treatment is an effective strategy to improve the electrocatalytic activity, but the improvement mechanism is still unclear. In this work, a nitrogen‐doped carbon nanotube‐encased iron nanoparticles (Fe@NCNT) catalyst is synthesized as the model catalyst, followed by plasma etching treatment with different parameters. The electrocatalytic activity improvement mechanism of the plasma etching treatment is revealed by combining the physicochemical characterizations and electrochemical results. As a result, highly active metal–nitrogen species introduced by nitrogen plasma etching treatment are recognized as the main contribution to the improved electrocatalytic activity, and the defects induced by plasma etching treatment also contribute to the improvement of the electrocatalytic activity. In addition, the prepared catalyst also demonstrates superior ORR activity and stability than the commercial Pt/C catalyst.

## Graphical abstract

This work studies the electrocatalytic activity improvement mechanism of the plasma etching treatment on oxygen reduction reaction catalysts, which reveals that the highly active metal‐nitrogen species induced by nitrogen plasma treatment is the main active contributor of the improved electrocatalytic activity.

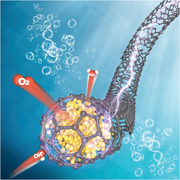

## 1 INTRODUCTION

Oxygen reduction reaction (ORR) plays an indispensable role in some renewable energy conversion and storage devices.^[^ ^1^, ^2^, ^3^, ^4^ ^]^ To date, Pt‐based catalysts are still considered as the best choice to drive the ORR at the cathode of the devices.^[^ ^5^, ^6^, ^7^, ^8^ ^]^ However, the low reserves and high price of the Pt metal seriously hinder its widespread commercial applications.^[^ ^9^, ^10^, ^11^ ^]^ Exploring high electrocatalytic activity and stability inexpensive catalysts to replace the Pt‐based catalysts has been one of the most pressing issues in driving commercial process of ORR‐relative renewable energy devices.^[^ ^12^, ^13^, ^14^ ^]^ Non‐noble metal catalysts, especially for the transition metal‐nitrogen‐carbon catalysts (M‐N‐C), are considered as one of the most promising alternatives to Pt‐based catalysts due to their low price and better intrinsic activity.^[^ ^15^, ^16^, ^17^ ^]^

Recently, the rational design and controllable preparation of M‐N‐C have been widely studied, and some inspiring results have been reported.^[^ ^18^, ^19^, ^20^, ^21^ ^]^ Several effective synthesis methods and activity improvement strategies have been proposed to optimize the electrocatalytic activity and stability of M‐N‐C catalysts.^[^ ^22^, ^23^, ^24^ ^]^ Among them, the plasma etching strategy has gained a lot of attention as a high‐efficient catalyst synthesis method in recent years, and also has proven to be an effective strategy to improve electrocatalytic performance through experimental researches.^[^ ^25^, ^26^, ^27^, ^28^ ^]^ However, the real electrocatalytic activity improvement mechanism of the plasma etching treatment is still not clear, which severely limits the research and application of plasma etching strategies in the controllable preparation of high‐efficient catalysts. Researchers usually only focus on analyzing the obtained isolated plasma treatment results, which makes our understanding of the electrocatalytic activity improvement mechanism of the plasma etching treatment rather one‐sided.

In this work, we have synthesized a nitrogen‐doped carbon nanotube encased iron nanoparticles catalyst as the model catalyst, and study the electrocatalytic activity improvement mechanism of the plasma etching treatment under different atmospheres with the same treatment conditions. The results reveal that the nitrogen plasma etching treated sample is significantly better than that of the argon treated catalysts. Combined with physicochemical characterizations and electrocatalytic data, the intrinsic reason for the improved electrocatalytic activity of the plasma‐treated catalysts can be attributed to the highly active metallic nitrogen species generated by the in situ nitrogen plasma etching treatment. In addition, the prepared catalyst also exhibits desirable ORR activity and zinc‐air battery's performance. This work not only reveals the electrocatalytic activity improvement mechanism of the plasma etching treatment, but also reports a high‐efficient ORR catalyst.

## 2 RESULTS AND DISCUSSION

**FIGURE 1:**
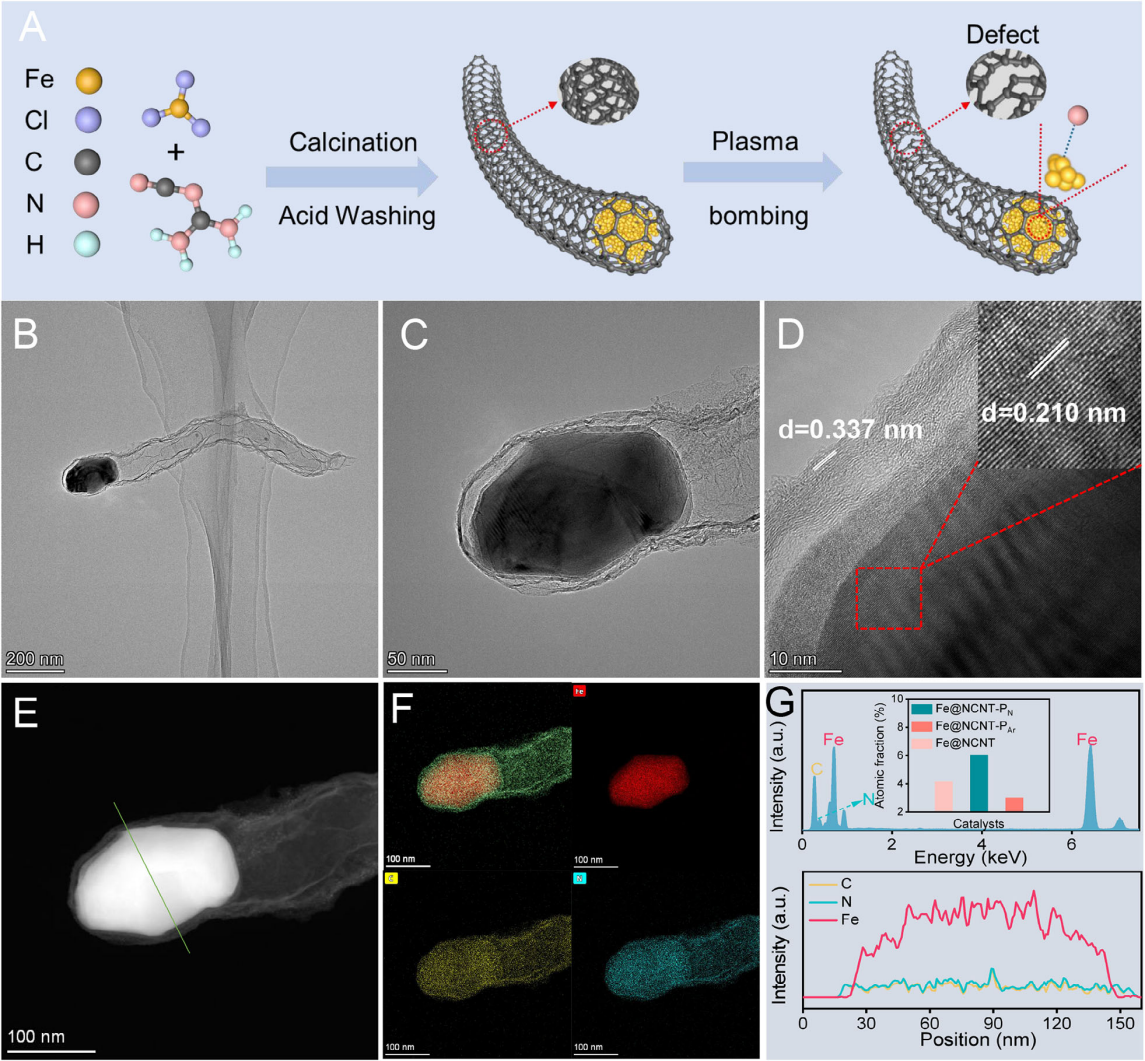
Morphology characterizations. A Schematic illustration of the prepared catalysts, B,C TEM, D HRTEM, E HAADF‐STEM and F EDX mapping images, and G EDS profile of Fe@NCNT‐P_N_.

The synthesis process of the prepared catalysts is schematically displayed in Figure 1A. Firstly, the metal precursor (FeCl_3_) and melamine are uniformly mixed and ground, followed by pyrolysis at 900°C for two hours under Ar atmosphere, and then remove the unstable active components from the obtained solid by acid washment (Fe@NCNT). Subsequently, Fe@NCNT is treated by plasma etching to prepare the final catalyst. The resultant catalysts prepared by the N_2_‐ and Ar‐plasma etching treatment are named as Fe@NCNT‐P_N_ and Fe@NCNT‐P_Ar_, respectively.

The scanning electron microscope (SEM) image reveals that Fe@NCNT exhibits a typical nanofiber nanostructure, and the length of the fiber is over 1 μm (Figure S1). In addition, there is a protruding sphere at the tip of each nanofiber, which probably covered with metal nanoparticles. The SEM images of Fe@NCNT‐P_N_ and Fe@NCNT‐P_Ar_ exhibit similar nanostructure with Fe@NCNT, indicating that the plasma etching treatment did not change the nanofiber structure of the prepared catalysts (Figures S2 and S3). The transmission electron microscope (TEM) images of the Fe@NCNT‐P_N_ demonstrate that the prepared catalysts exhibit a hollow nanotube nanostructure, and the protruding sphere at the tip of each nanotube is the metallic nanoparticles, showing the typical core–shell nanostructure (Figure 1B,C). In addition, the formation mechanism of resultant nanotube is that rearrangement and curling of the carbon atoms to form the typical nanotube nanostructure with catalyzed by the iron species. There are two obvious lattice fringes are observed in the high‐resolution TEM (HRTEM) image of the Fe@NCNT‐P_N_, with a d‐spacing of 0.337 and 0.210 nm, corresponding to the (111) plane of the graphitic carbon (PDF # 41–1487) and (111) plane of Fe (PDF #50‐1275), respectively (Figure 1D). The TEM energy dispersive X‐ray spectroscopy (EDX) mapping images demonstrate that carbon (yellow) and N (blue) are homogeneously distributed over the whole supports of Fe@NCNT‐P_N_, and the Fe metallic (red) nanoparticles are well encased in the carbon shell (Figures 1E,F). The line‐scanning analysis of Fe@NCNT‐P_N_ demonstrates that the Fe and N elements are dispersed uniformly across the whole core–shell supports with an *N* content of 6.01% (Figure 1G). Moreover, the micromorphology of Fe@NCNT, Fe@NCNT‐P_N_, and Fe@NCNT‐P_Ar_ catalysts is consistent, indicating that the plasma etching did not change the microstructure of the material (Figure 1B,C, Figures S4 and S5). The *N* content is the only changed signal after the plasma etching treatment, the *N* content of Fe@NCNT‐P_N_ is significantly increased relative to the other two catalysts, which could be owing to the nitrogen doping induced by the N_2_ plasma etching treatment (Figure 1G, Figures S4 and S5).

**FIGURE 2:**
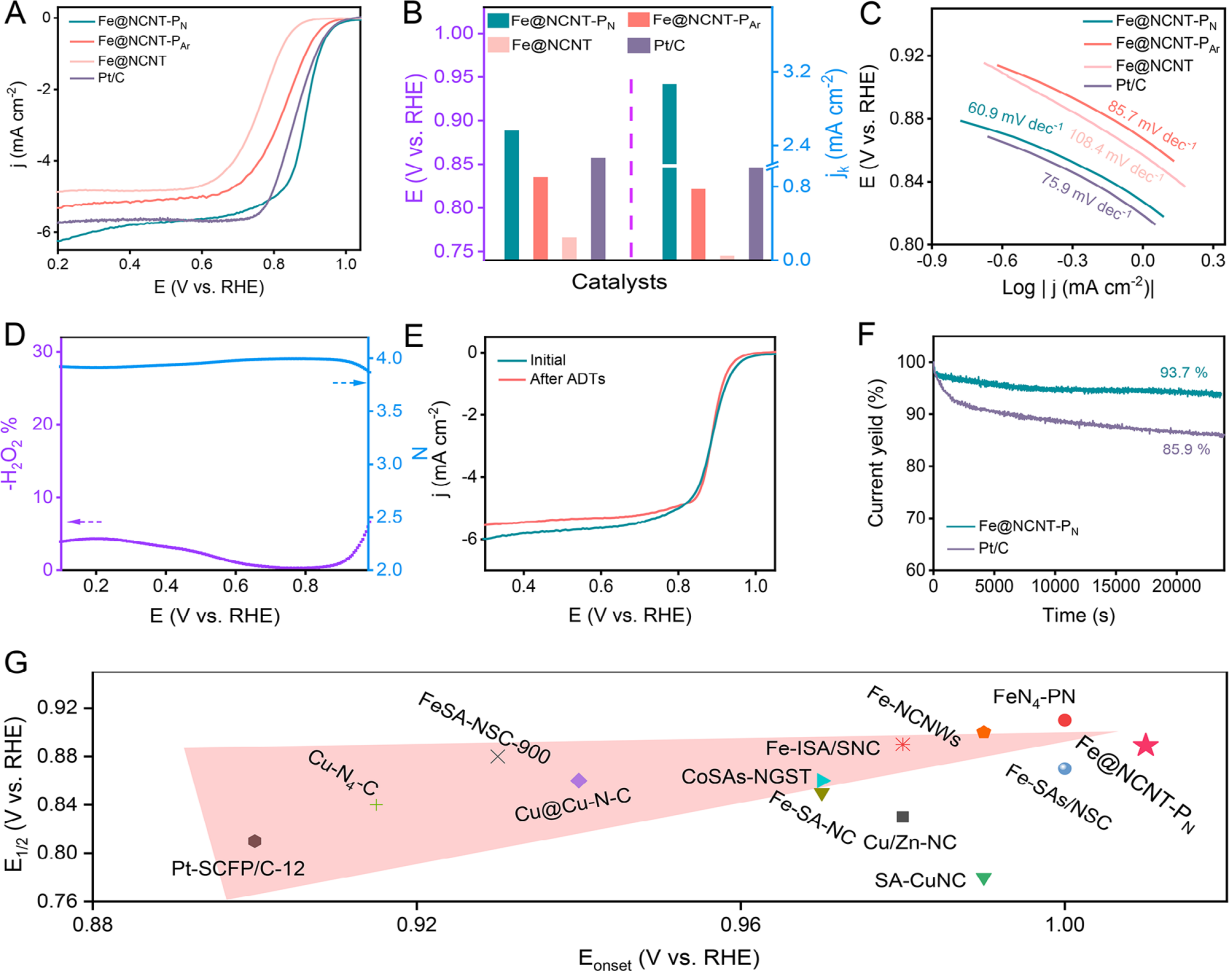
ORR performance of the prepared catalysts. A LSV curves and B comparison of the half‐wave potential and kinetics current density, and C Tafel slope of the prepared catalysts and Pt/C, D RRDE result of the Fe@NCNT‐P_N_, E LSV curves of the Fe@NCNT‐P_N_ before and after ADT, F CA results of Fe@NCNT‐P_N_ and Pt/C, G comparison of ORR performance of prepared catalysts and recently reported catalysts.

The electrochemical performance of the prepared catalysts is evaluated by a three‐electrode system in an alkaline medium. The ORR performance of the commercial Pt/C is measured by the same conditions as the benchmark. The linear sweep voltammetry (LSV) curves of the Fe@NCNT‐P_N_ and Fe@NCNT‐P_Ar_ are significantly positive‐shifted compared with the pristine Fe@NCNT, suggesting that proper plasma etching treatment can indeed enhance the electrocatalytic activity of the catalysts (Figure 2A). In addition, the electrocatalytic activity of the nitrogen plasma etching treated catalyst (Fe@NCNT‐P_N_) is significantly superior to that of the argon treated catalyst (Fe@NCNT‐P_Ar_), with a higher kinetic current density and half‐wave potential of 3.064 mA cm^−2^ @ 0.9 V and 0.889 (Figure 2B). The conditions for both nitrogen and argon plasma etching treatment are similar, confirming that the nitrogen plasma etching treatment has a stronger ability to improve electrocatalytic activity. Moreover, the ORR activity of Fe@NCNT‐P_N_ is much better than Pt/C, in terms of the half‐wave potential (0.889 vs 0.857 V) and kinetic density (3.064 vs. 1.759 mA cm^−2^) (Figure 2B). The Tafel slope of Fe@NCNT‐P_N_ is 60.9 mV dec^‐1^, which is much lower than Pt/C (75.9 mV dec^‐1^) and Fe@NCNT‐P_Ar_ (85.7 mV dec^‐1^), confirming the nitrogen plasma etching treatment indeed improve the electrocatalytic performance (Figure 2C). The electron transfer number of the prepared catalysts has been calculated by rotating ring disk electrode (RRDE), confirming that Fe@NCNT‐P_N_ has a typical 4‐electron dominated ORR process (Figure 2D).

The stability and durability of Fe@NCNT‐P_N_ catalyst are also measured via a three‐electrode system. The LSV curves of the Fe@NCNT‐P_N_ before and after ADT indicate that almost no loss of catalytic activity after 20000 cycles ADT test (Figure 2E). The chronoamperometry (CA) is also conducted to test the stability of Fe@NCNT‐P_N_ and Pt/C. The current density retention of Fe@NCNT‐P_N_ is 93.7% after the CA test for more than 24,000 s, while it's 85.9% for Pt/C under the same condition (Figure 2F). In addition, the ORR activity of the prepared catalysts and recently reported data are summarized in Figure 2G and Tables S1, which confirms Fe@NCNT‐P_N_ delivers a competitive ORR performance among all the collected data.

**FIGURE 3:**
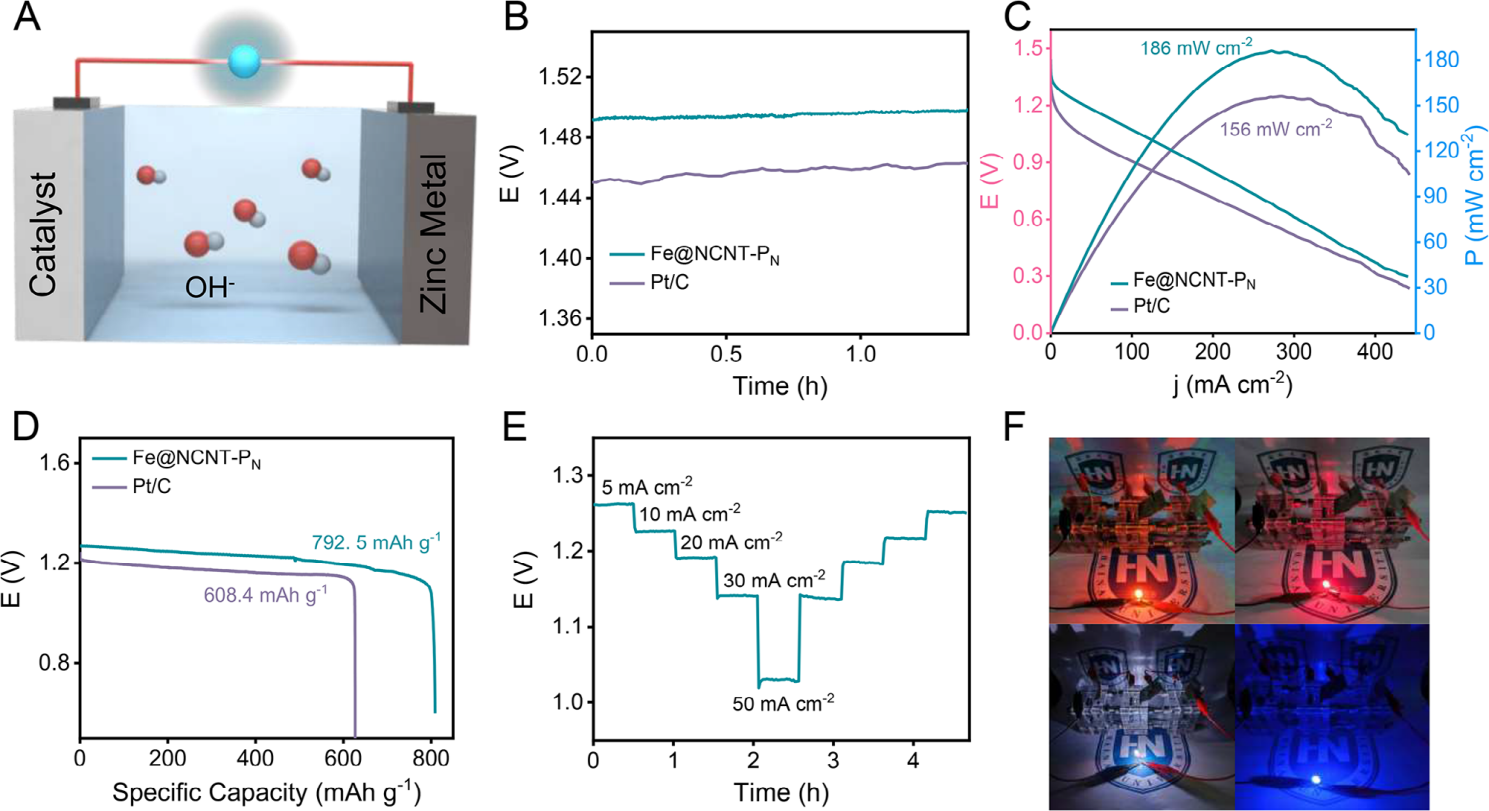
ZAB performance of the assembled ZAB. A Schematic structure of ZAB, B OCP, C polarization and power curves, D specific capacity of the Fe@NCNT‐P_N_ and Pt/C, E rate‐discharge curves of the Fe@NCNT‐P_N_, F three ZAB connected in series to power LED light.

Fe@NCNT‐P_N_ is also assembled into a zinc‐air battery (ZAB) to verify their electrocatalytic performance in the practical devices. In the assembled ZAB, the polished Zinc plate is the anode electrode, the carbon coated Fe@NCNT‐P_N_ catalysts are the cathode electrode, and the 6 m KOH is the electrolyte (Figure 3A). To simplify the name of the assembled ZAB, the ZAB assembled with Pt/C and Fe@NCNT‐P_N_ as cathode catalysts are denoted as the Pt/C and Fe@NCNT‐P_N_, respectively. The open circle potential (OCP) of the Fe@NCNT‐P_N_ is 1.50 V, which is 40 mV higher than Pt/C, suggesting a better battery performance of Fe@NCNT‐P_N_ (Figure 3B). In addition, the Fe@NCNT‐P_N_ delivers a high discharge ability, with a high peak power density of 186 mW cm^−2^, and the peak power density of the Pt/C is only 156 mW cm^−2^ (Figure 3C). The specific capacity of Pt/C and Fe@NCNT‐P_N_ is 608.4 and 792.5 mAh g^‐1^, respectively, which provides another evidence of the good electrocatalytic activity of Fe@NCNT‐P_N_ (Figure 3D). Moreover, the assembled ZAB also shows s stable discharge current stage at a current density range from 5 to 50 mA cm^−2^, and the battery voltage can be well recovered when the current density is reversed back to initial data (Figure 3E). The assembled ZAB contact in series can easily power the LED light, indicating the potential application of the prepared catalysts (Figure 3F). Moreover, the assembled ZAB also exhibits competitive battery performance among the recently reported works (Table S2). There all electrochemical data reveal that the nitrogen plasma etching treatment is superior to argon treatment for improving the electrocatalytic activity, and Fe@NCNT‐P_N_ delivers a desirable ORR activity in terms of both three‐electrode system and ZAB level.

**FIGURE 4:**
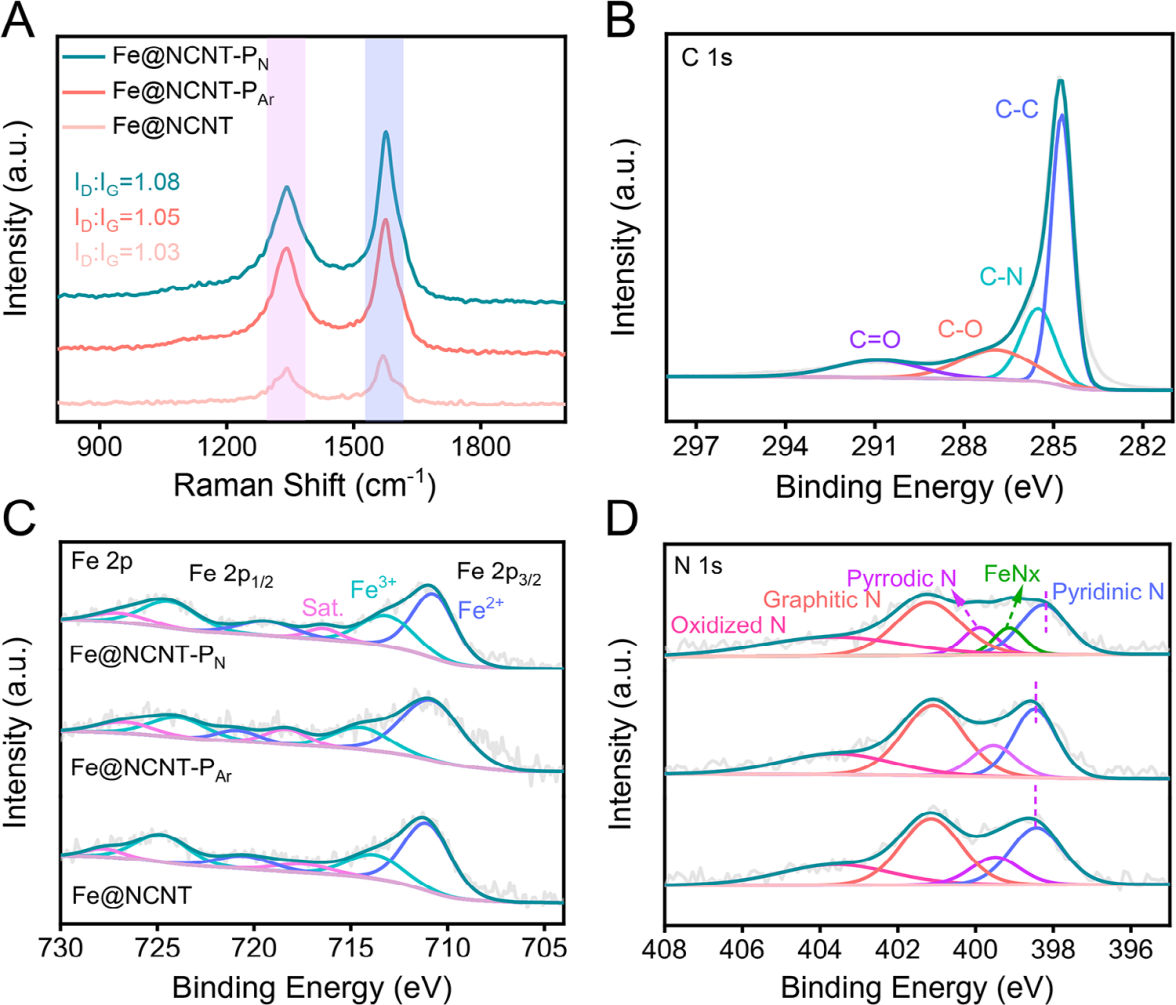
Characterizations of the prepared catalysts. A Raman spectra of the prepared catalysts, B C 1s high‐resolution XPS peak of the Fe@NCNT‐P_N_, C Fe 2p and D N 1s high‐resolution XPS peaks of the Fe@NCNT, Fe@NCNT‐P_N_, and Fe@NCNT‐P_Ar_.

To further determine the real electrocatalytic performance improvement mechanism of the plasma etching treatment, more physicochemical characterizations are carried out on the nitrogen and argon plasma etching treated catalysts (e.g. Fe@NCNT‐P_N_ and Fe@NCNT‐P_Ar_). In general, plasma etching treatment has been believed to in situ construct defects on the catalysts, and thus improve the electrocatalytic activity.^[^ ^28^, ^29^, ^30^ ^]^ The D band and G band of the Raman spectrum are considered to correlate with the degree of graphitization and defects, respectively, and the ratio of *I_D_* and *I_G_* can qualitatively analyze the degree of defects in carbon materials. The *I_D_*:*I_G_* value of the plasma etching treated catalysts is much higher than that of the pristine catalysts, confirming the plasma etching treatment has indeed induced more defects on the carbon supports (Figure 4A).^[^ ^31^ ^]^ Moreover, the *I_D_*:*I_G_* value of Fe@NCNT‐P_N_ and Fe@NCNT‐P_Ar_ is 1.08 and 1.05, respectively, suggesting the nitrogen plasma etching treatment is more effective for manufacturing defects (Figure 4A). C 1s high‐resolution XPS peak of Fe@NCNT‐P_N_ could be deconvolved into four types peaks, corresponding to C─C, C─N, C─O, C═O (Figure 4B).^[^ ^32^, ^33^ ^]^ Fe 2p high‐resolution XPS peaks of the prepared catalysts could be well fitted with Fe^2+^, Fe^3+^ and satellite species (Figure 4C).^[^ ^34^, ^35^ ^]^ It is noted that the XPS signals after plasma etching treatment are shifted toward lower binding energy, indicating the valence state of the catalyst has a little decrease after plasma etching treatment. N 1s high‐resolution XPS peaks have been deconvolved into four types of peaks, assigning to the Oxidized N, Graphitic N, Pyrrolic N, and Pyridinic N (Figure 4D).^[^ ^36^, ^37^, ^38^ ^]^ There is a new peak is observed on the N 1s high‐resolution XPS peak of Fe@NCNT‐P_N_, it could be assigned to Fe‐N species.^[^ ^39^ ^]^ The recently reported works have confirmed that the Fe‐N species are the highly active centres towards ORR.^[^ ^40^, ^41^, ^42^ ^]^

Combined with the electrochemical data and physicochemical characterization results, we think that the electrocatalytic activity improvement mechanism of the plasma etching treatment should be divided into two parts, (1) the defects induced by plasma etching (including the nitrogen and argon plasma etching treatment), (2) the highly active metal‐nitrogen species induced by nitrogen plasma treatment, which is the main active contributor of the improved electrocatalytic activity.

## 3 CONCLUSION

In conclusion, we have applied the plasma etching technology to treat the model catalysts to investigate the effect of different plasma etching treatment on the electrocatalytic activity, and further analyze the electrocatalytic activity improvement mechanism through combination with the characterization results and electrocatalytic data. Plasma etching treatment induced defects and metal‐nitrogen species both improve the electrocatalytic performance, and the metal‐nitrogen species are the main contributor. Moreover, the prepared catalyst exhibits excellent ORR performance, which is superior to Pt/C in both three‐electrode systems and ZAB levels. This work reveals the real mechanism of plasma etching treatment to improve the electrocatalytic activity, it also provides new ideas for the development of synthesis strategy of high‐efficient catalysts.

## CONFLICT OF INTEREST

The authors declare no conflicts of interest.

## Supporting information

Supplementary Information

## Data Availability

The data are available on request from the authors.
