# Supplementary material for: Understanding the improvement mechanism of plasma etching treatment on oxygen reduction reaction catalysts
Source: Exploration (Beijing). 2023 Nov 14;4(1):20230034. doi: 10.1002/EXP.20230034 (PMC10867369; doi:10.1002/EXP.20230034)
Supplement: Supplementary file 1 — Supplementary Information [file EXP2-4-20230034-s001.docx]

**Understanding the improvement mechanism of plasma etching treatment on oxygen reduction reaction catalysts**

Peng Rao ^a, #^, Yanhui Yu ^a, #^, Shaolei Wang ^b,^ *, Yu Zhou ^a^, Xiao Wu ^c^, Ke Li ^c^, Anyuan Qi ^c^, [Peilin Deng](https://pubs.rsc.org/en/results?searchtext=Author%3APeilin%20Deng) ^a^, Yonggang Cheng ^d^, [Jing Li](https://pubs.rsc.org/en/results?searchtext=Author%3AJing%20Li) ^a^, Zhengpei Miao ^a^, and Xinlong Tian ^a,^*

^a^ School of Marine Science and Engineering, Hainan Provincial Key Lab of Fine Chemistry, School of Chemical Engineering and Technology, Hainan University, Haikou, 570228, China

^b^ Key Laboratory of Polyoxometalate and Reticular Material Chemistry of Ministry of Education, School of Chemistry, Northeast Normal University, Changchun 130024, China

^c^ National Energy Group Ledong Power Generation Co., Ltd, Ledong 572539, China

^d^ Laboratory for chemical technology, Ghent University, Technologiepark 125, 9052 Gent, Belgium

^#^ P. Rao and Y. Yu contributed equally to this work.

^*^***Corresponding authors****:* *Shaolei Wang* (*wangsl030@nenu.edu.cn), Xinlong Tian (tianxl@hainanu.edu.cn)*

**Methods**

**Chemicals**

Ferric chloride hexahydrate (FeCl_3_·6H_2_O), Dicyandiamide (C_2_H_4_N_4_), Hydrochloric acid (HCl), and KOH purchased from Shanghai Macklin Biochemical Co., Ltd. 20% Pt/C was obtained from Johnson Matthey (JM) Corp. Nafion solution (5 wt.%) were obtained from DuPont. All chemicals were used without further purification. All aqueous solutions were prepared using deionized (DI) water with a resistivity of 18.2 MΩ.

Synthesis of Fe@NCNT

Firstly, 0.452g of FeCl_3_·6H_2_O and 0.864g of C_2_H_4_N_4_ were ground and mixed well. The mixed samples were then placed in a tube furnace and calcined at high temperature under Ar gas atmosphere. After cooling to room temperature, the obtained product was placed in 1.0 M HCl and acid-washed for 24 h to obtain Fe@NCNT. The most suitable precursors were selected by regulating the ratio of FeCl_3_·6H_2_O.

Synthesis of Fe@NCNT-P_N_

The acid-washed sample were placed into the middle of the plasma enhanced chemical vapor deposition (PECVD) in sequence, and then started on the tube furnace of the PECVD with the following parameters, the temperature was 300℃, holding time was 60 min, and under the N_2_ flowing. When the temperature reached the setting value, turn on the PECVD with the following parameters, radio frequency (RF, 13.56 MHz) power was 300 W, the processing time was 30 min, the tube pressure was 50 Pa, and under the N_2_ flowing. After the temperature of the tube drops to room temperature, the Fe@NCNT-P_N_ was obtained.

Synthesis of Fe@NCNT-P_Ar_

To further investigate the effect of different atmospheres on the catalyst performance, the N_2_ in PECVD was changed to Ar and other parameters were kept constant. After the temperature of the tube drops to room temperature, the Fe@NCNT-P_Ar_ was obtained.

**Material characterizations**

The TEM and HRTEM were tested by using a Thermo Scientific Talos F200X G2 operated at 200 keV. XRD was conducted on an HAOYUAN powder diffractometer (DX-2700BH). X-ray photoelectron spectroscopy (XPS) was performed on a Thermo ESCALAB 250XI photoelectron spectrometer employing a monochromated Al-K Xray source (hν = 1486.6 eV). Raman spectra were recorded at ambient Horiba Scientific Raman Spectrometer using the exciting line at 532 nm of a diode Laser.

**Electrochemical measurements**

In brief, the ORR performance tests were conducted on Multi autolab M240 electrocatalytic station. A glass carbon electrode (GCE), a carbon rod, and a Hg/HgO was applied as working electrode, counter electrode, and reference electrode, respectively. The electrolyte was composed of the 0.1 M KOH. The catalysis ink was prepared via uniformly disperse of 5mg catalysts, 970 μL of ethanol and 30 μL Nafion. After that, drop 7 μL of the catalysis ink onto the GCE to obtain the testing working electrode. The LSV curves were recorded at 1600 revolutions per minute (rpm) with a scan rate of 5mV·s^−1^ in O_2_-saturated 0.1M KOH. The accelerated durability test (ADT) method was applied to test the durability, with a 20,000 cycles potential cycling from 0.6 to 1.0 V at 100 mV s^−1^. The stabilities of the catalyst were studied by chronoamperometry (CA) at 0.6 V vs. RHE in an O_2_-saturated electrolyte.

Rotating ring-disk electrode (RRDE) measurements of the samples was measured to study the four-electron selectivity. The Pt ring electrode was biased at 1.1 V vs. RHE. The H_2_O_2_ yield and n per oxygen molecule were calculated by the following equations:

$\%H_{2}O_{2}=200\frac{I_{R}/N}{I_{D}+I_{R}/N}$ (1)

$n=4\frac{I_{D}}{I_{D}+I_{R}/N}$ (2)

where I_D_ and I_R_ are the disk and ring currents, respectively. N is the ring current collection efficiency (37%).

Kinetic current density (j_k_) of the prepared catalysts was calculated via the Koutechy-Levich (K-L) function ^1^:

$\frac{1}{J}=\frac{1}{J_{k}}+\frac{1}{J_{L}}$ (1)

J: Cathodic electrode current

J_k_: Kinetic current density

J_L_: Limiting current density

Tafel slope measurement was calculated based on Tafel function^2^:

η= a + b lgi (2)

in which, η = U - U_0_, b = 2.3RT/(αF), η is the overpotential, i is the current density, a is the transfer number, b is Tafel slope, R is the gas constant, T is the temperature, and F is the Faraday constant.

All potentials in this work are quoted with respect to a reversible hydrogen electrode (RHE). The potentials in LSV curves in this study have subjected to iR compensation.

**Zinc-Air Battery (ZAB) Test**

The anode was polished Zinc plate, cathode was carbon paper loaded catalysts, and the electrolyte was 6 mol∙L^−1^ KOH. The polarization curves were recorded by the Multi Autolab M240 electrochemical workstation. The LANHE (CT2001A) station was applied to test the discharge polarization curve of the assembled ZAB.


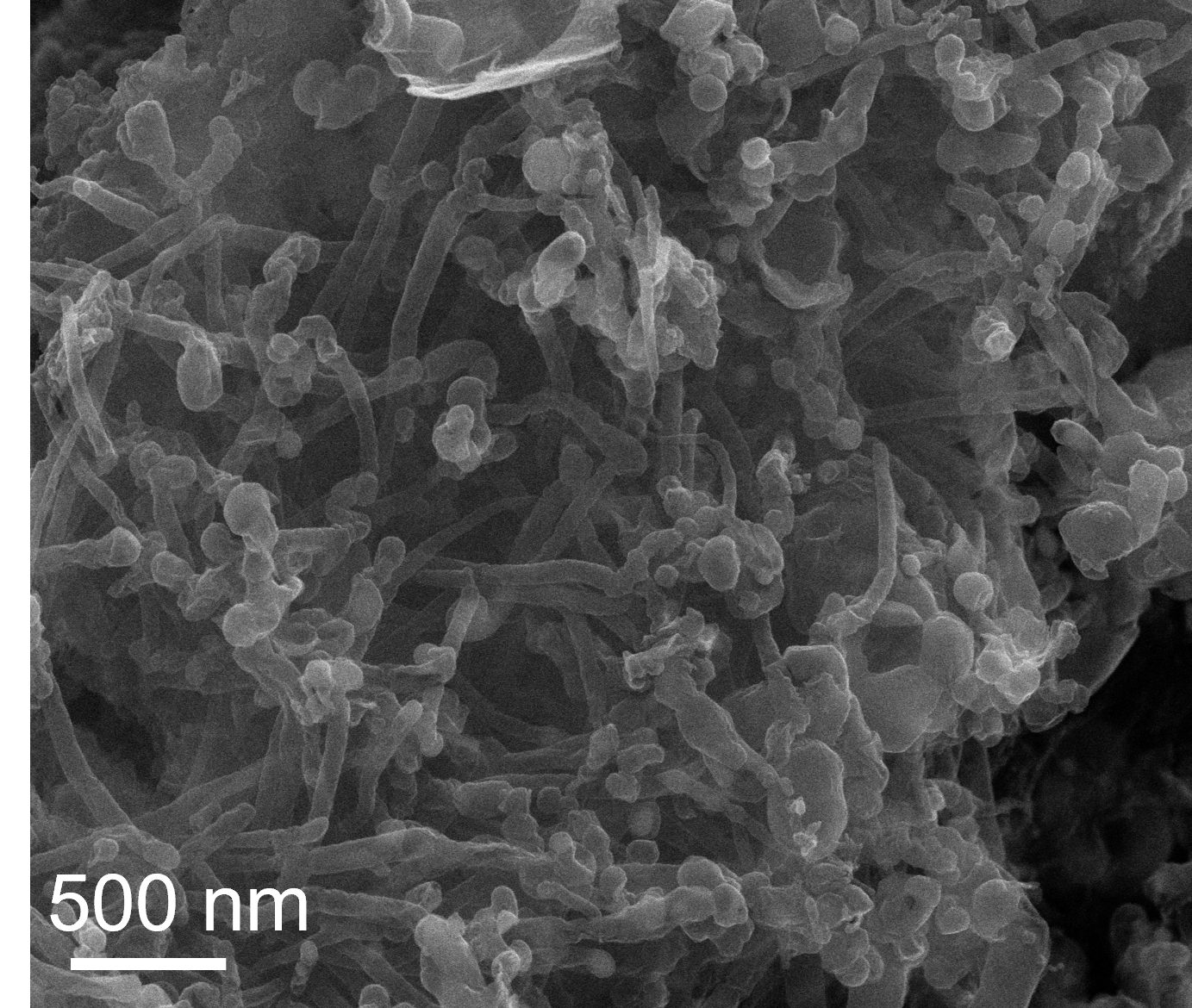


**Figure S1** SEM image of the prepared Fe@NCNT.


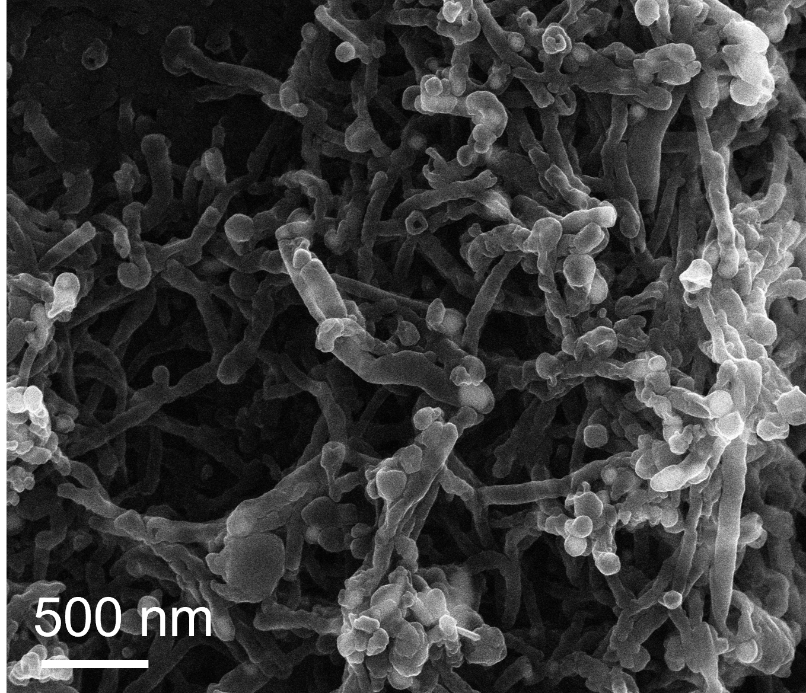


**Figure S2** SEM image of the prepared Fe@NCNT-P_N_.


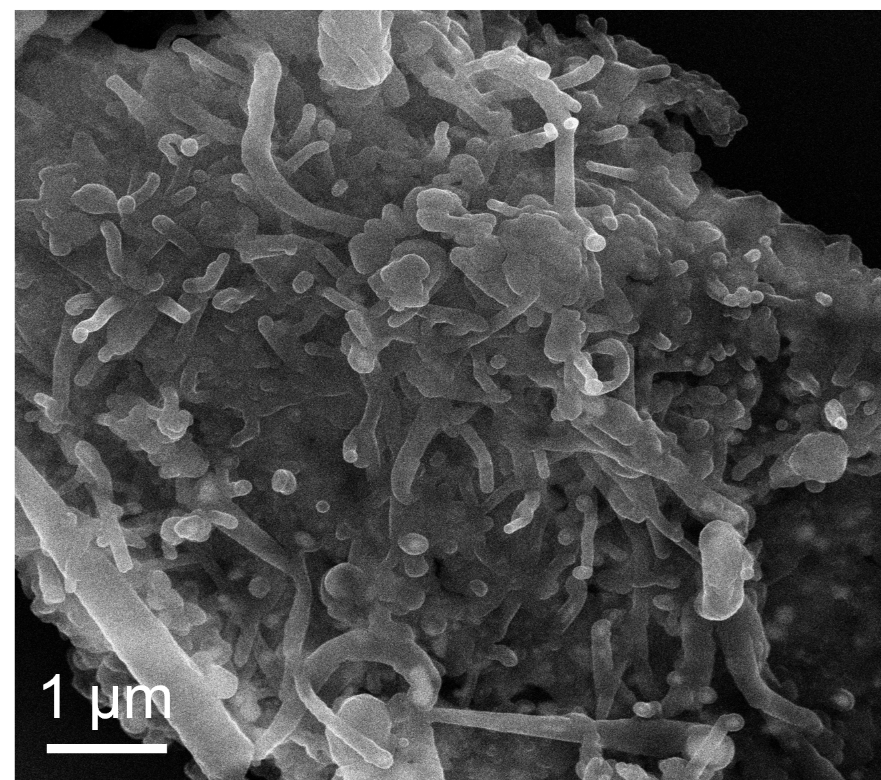


**Figure S3** SEM image of the prepared Fe@NCNT-P_Ar_.


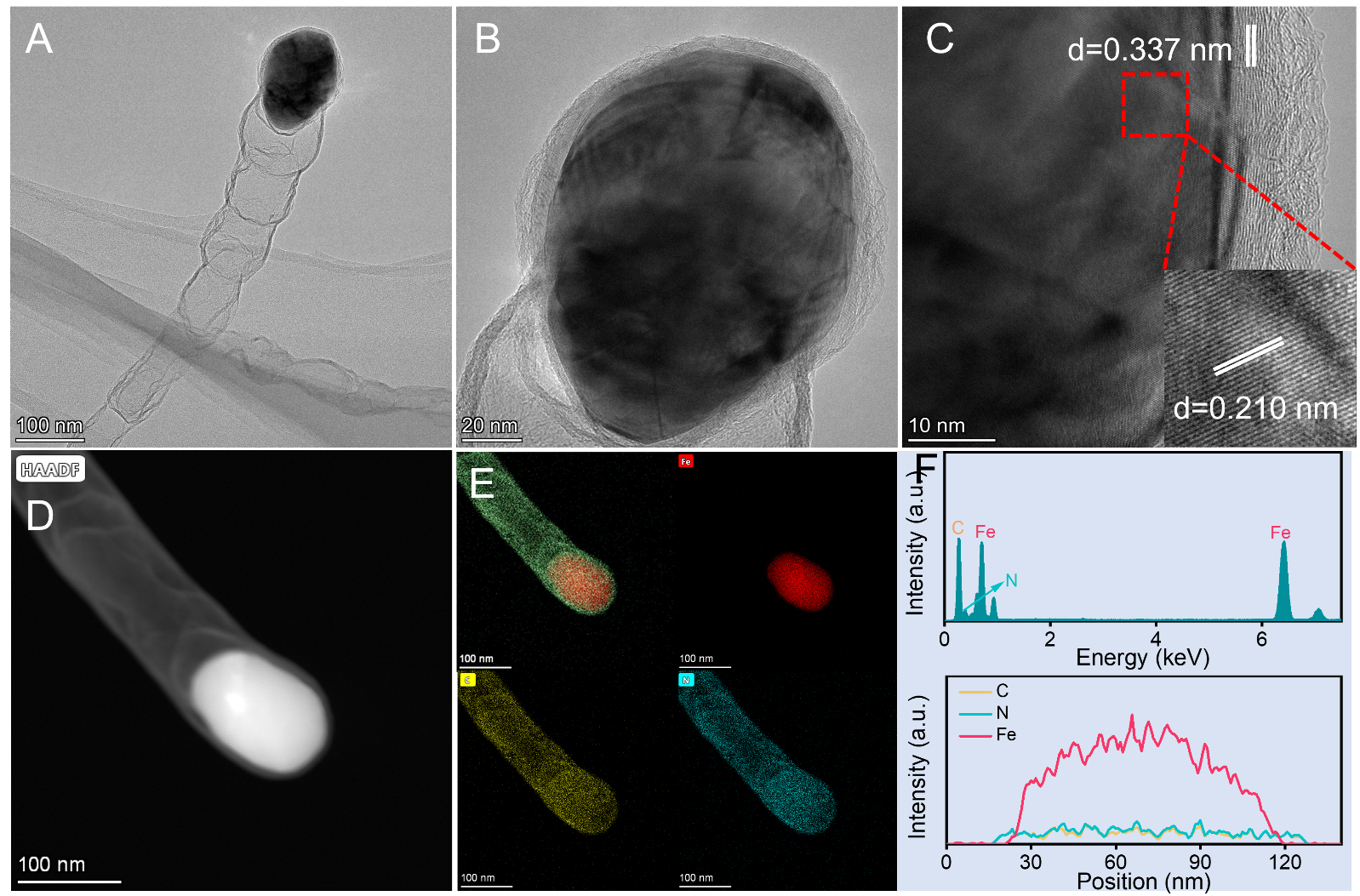


**Figure S4** Microstructural properties. **(A-B)** TEM, **(C)** HRTEM, **(D)** HAADF-STEM and **(E)** EDX mapping images, and **(F)** EDS profile of the prepared Fe@NCNT catalysts.


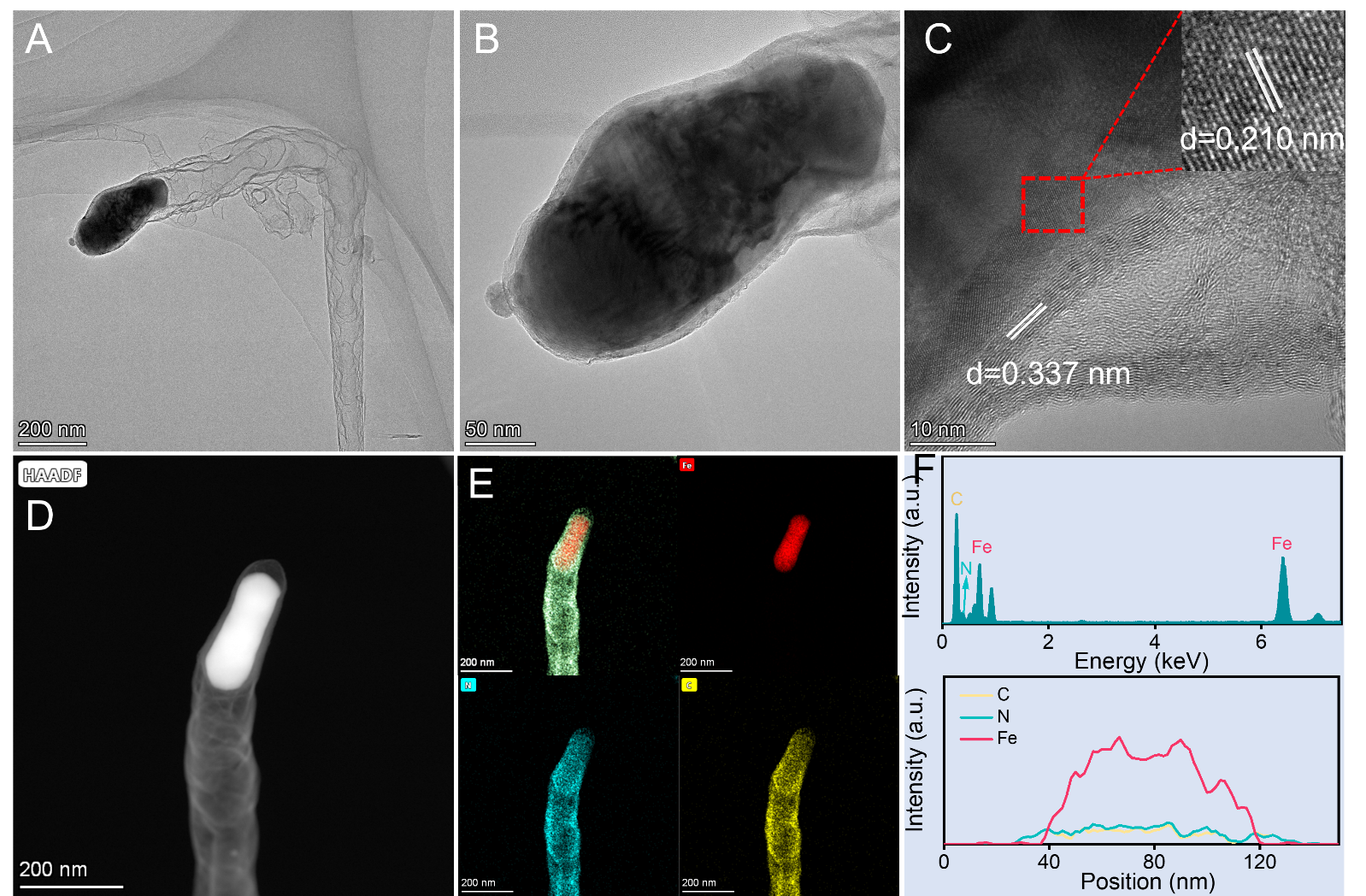


**Figure S5** Microstructural properties. **(A-B)** TEM, **(C)** HRTEM, **(D)** HAADF-STEM and **(E)** EDX mapping images, and **(F)** EDS profile of the prepared Fe@NCNT-P_Ar_ catalyst.

**Table S1** Comparation of the ORR performance of the prepared catalyst and recently reported catalysts.

| Catalysts | E_ONSET_  V vs. RHE | E_1/2_  V vs. RHE | References |
| --- | --- | --- | --- |
| **Fe@NCNT-P_N_** | **0.98** | **0.889** | **This work** |
| Cu/Zn-NC | 0.98 | 0.83 | *Angew. Chem. Int. Ed.*, **2021**, 60, 14005. |
| FeN_4_-PN | 1.0* | 0.91 | *ACS Catal*., **2021**, 11, 6304. |
| CoSAs-NGST | 0.99 | 0.89 | *Adv. Funct. Mater.*, **2021**, 31, 2010472. |
| SA-CuNC | 0.99* | 0.78 | *Adv. Energy Mater.*, **2021**, 11, 2100303. |
| Fe SA-NSC-900 | 0.94 | 0.86 | *ACS Energy Lett.*, **2021**, 6, 379. |
| Pt-SCFP/C-12 | 0.90 | 0.81 | *Adv. Energy Mater.*, **2020**, 10, 1903271. |
| Cu@Cu-N-C | 0.97 | 0.85 | *Small* **2019**, 15, 1902410. |
| Fe-NCNWs | 0.99 | 0.90 | *ACS Catal.*, **2019**, 9, 5929. |
| Fe-SAs/NSC | 1.00 | 0.87 | *J. Am. Chem. Soc.*, **2019**, 141, 20118. |
| Cu-N_4_-C | 0.915 | 0.84 | *ACS Nano* **2019**, 13, 3177. |
| Fe-ISA/SNC | 0.98 | 0.89 | *Adv. Mater.*, **2018**, 30, e1800588. |
| Fe-SA-NC | 1.02* | 0.902 | *J. Mater. Chem. A*, **2023**,11, 5288. |

NOTE: *The data is not given, but excavated from the LSV curves.

**Table S2** Comparison of the ZAB performance of the assembled ZAB and recently reported ZABs.

| Catalysts | Power density (mW cm^-2^) | Specific capacity (mAh g^-1^) | References |
| --- | --- | --- | --- |
| **Fe@NCNT-P_N_** | **186** | **792.5** | **This work** |
| FeCoNiMoW | 116.9 | 857 | *Adv. Mater.*, **2023**, [10.1002/adma.202303719](https://doi.org/10.1002/adma.202303719). |
| SA-Co-N_4_-GCs | 149.3 | 764.8 | *Nano Res.*, **2022**, 15, 7209. |
| PtFe-HNC-C | 159.6 | 710.5 | *Carbon Energy*, **2022**, 4, 1003. |
| NiFe/NCNF/CC | 140.1 | 730 | *Appl. Catal. B: Environ.*, **2021**, 285, 119856. |
| N, Co-CNTs | 114 | - | *Appl. Catal. B: Environ.*, **2021**, 283, 119643. |
| Ni@N-HCGHF | 117.1 | 706 | *Adv. Mater.*, **2020**, 32, 2003313. |
| Co_4_N@NC-2 | 74.3 | 769.4 | *Appl. Catal. B: Environ.*, **2020**, 275, 119104. |

**Reference**

1. S. Treimer, A. Tang, D.C.A. Johnson, *Electroanalysis* **2002**, *14*, 165-171.

2. J. Tafel, *Zeitschrift für Physikalische Chemie*, **1905**, *50U*, 641-712.
